# Impaired Motor Function in the Affected Arm Predicts Impaired Postural Balance After Stroke: A Cross Sectional Study

**DOI:** 10.3389/fneur.2019.00912

**Published:** 2019-08-21

**Authors:** Lena Rafsten, Christiane Meirelles, Anna Danielsson, Katharina S. Sunnerhagen

**Affiliations:** ^1^Department of Clinical Neuroscience and Rehabilitation Medicine, Institute of Neuroscience and Physiology, University of Gothenburg, Sahlgrenska Academy, Gothenburg, Sweden; ^2^Centre for Person-Centred Care (GPCC), University of Gothenburg, Gothenburg, Sweden; ^3^Department of Therapy Service, University of Chicago Medical Center, Chicago, IL, United States; ^4^Department of Health and Rehabilitation, Institute of Neuroscience and Physiology, University of Gothenburg, Sahlgrenska Academy, Gothenburg, Sweden

**Keywords:** stroke, arm, postural balance, upper extremity, outcome measure

## Abstract

**Background:** Impaired postural balance is a common symptom after stroke and a common cause of falling. Most common daily tasks use arm and hand movements. Impairment in an upper extremity is a common stroke symptom, affecting 50–80% in the acute phase after stroke, and 40–50% in the sub-acute phase. The impact of leg function on postural balance has been investigated in several studies, but few have stressed the importance of arm function on postural balance.

**Objective:** To explore whether there is any association between arm function and postural balance after stroke.

**Method:** A cross sectional study where 121 adults (mean age: 70 ± 12.3 years, 72 men) from two different data sources, Gothenburg Very Early Supported Discharge (GOTVED), and a study by Carvalho et al. were merged. Time for assessments ranged from 1 to 13 years when the patients were in the chronic phase. The dependent variables were Berg Balance scale (BBS) and Time Up and Go (TUG) both dichotomized to “impaired postural balance” and “not impaired postural balance.” As independent variables, the Fugl-Meyer Assessment-Upper Extremity (FMA-UE) scale was used. The FMA-UE was presented with the total score.

**Results:** The motor function in the arm affected after stroke onset correlated with postural balance both measured with the BBS (0.321, *p* < 0.001*)* and the TUG (−0.315, *p* = 0.001*)*. Having impaired motor function in the arm was significantly associated with impaired postural balance assessed with the BBS with OR = 0.879 (CI 0.826–0.934, *p* < 0.001). Regression analysis with the TUG showed the same result, OR = 0.868 (CI 0.813–0.927, *p* < 0.001) for FM-UE.

**Conclusion:** The motor function of the affected arm was significantly associated with impaired postural balance post stroke, as assessed by BBS or TUG. It could be of clinical importance to be aware of the fact that not only lower extremity impairment, but also arm function can have an impact on postural balance in a late stage after stroke.

**Trial Registration:** VGFOUGSB-669501.

## Introduction

Every year about 25 million people are diagnosed with stroke (1–3), and six and a half million die from stroke (4, 5). A majority of those who survive a stroke experience a combination of loss of: muscle strength, sensation, balance, cognition, and emotion leading to restrictions in their ability to perform activities of daily living (ADL).

Postural balance is the ability to control the center of mass (COM) in the relation to the base of support (BOS) (6). Postural balance is essential for optimal performance of many daily activities (7). Impaired postural balance is a common symptom after stroke and a common cause of falling (8, 9). Falls occur at all stages post stroke but the risk of falling is even higher at later stage after stroke compared to similarly aged individuals (10–13). It has been shown that the risk of falling was more than two times higher in those with stroke compared with age- and gender-matched controls (14). Impaired motor- and sensory function are probably the two consequences that have the greatest impact on postural balance and on walking ability after stroke (15).

Arm and hand movements are used in most common daily tasks (6). Impairment in an upper extremity is a common symptom that affects 50–80% in the acute phase after stroke (16, 17), and 40–50% in the sub-acute phase (7, 18). Studies have shown that the “not affected” arm is also affected after a stroke (19). The effects of arm swing on gait stability are uncertain, but it is clear that arms may help in regaining postural balance after has been perturbed, and that safe positions may be adopted by those at risk of falling (20). One such study observed that asymmetrical muscle function in the shoulder contributes to the recovery of postural balance after a person has stumbled and is about to fall (21). Several studies show that leg function impacts postural balance, but few studies have shown the importance of arms on postural balance (22). This is why further studies are needed.

The aim of this study was to explore if there is any association between arm function and postural balance in a late stage after stroke.

## Materials and Methods

### Design

A cross sectional design with data from persons with chronic stroke was used (23). The Strengthening the Reporting of Observational Studies in Epidemiology (STROBE) statements for observational studies was followed.

### Settings and Participants

Data were retrieved from two different data sources, Gothenburg Very Early Supported Discharge (GOTVED) (24), and a study by Carvalho et al. (25) and merged into one dataset for analysis. In order to obtain a larger sample, we found it rational to merge these data as they are homogeneous in terms of age, gender, diagnosis and measurements. All measurements in the different data sources were also made during the chronic phase after stroke. Inclusion criteria were confirmed stroke according to World Health Organization (WHO) criteria (26), age ≥18 years, and having been assessed with Berg Balance Scale (BBS), Time Up and Go (TUG) and Fugl-Meyer Assessment (FMA). The Regional Ethics Examination Board in Gothenburg (042-11, 426-05) approved this study. Written informed consent was obtained from the participants or from their closest relative prior to participation in any of the studies.

### Outcome Measures

The dependent variable was postural balance as assessed by BBS (27–29), and TUG (30), each used in different regression models. BBS has been shown to be an appropriate screening tool to predict fall risk at a moderate accuracy level (31). When dichotomising BBS we chose a BBS < 45 to identify impaired postural balance (29, 32). The TUG is commonly used to examine functional mobility, reflecting postural balance and gait maneuvers used in daily life (30). TUG was performed twice and the second value was used. Use of walking aids was registered. In the present study, we adopted a TUG cut-off score >15 s (32, 33) to identify impaired postural balance.

The independent variable was motor function, as assessed by Fugl-Meyer Assessment (FMA) (34) divided into upper extremity (UE) and lower extremity (LE) sections. The FMA has been found to be reliable and valid for this group (34–36). The FMA was presented with the total score, and in the two different sections (FMA-UE) consisting of 33 items, each item scored 0–2 and summed to a total score of 0–66 points, and Lower Extremity (FMA-LE), consisting of 17 items and summed to a total score of 0–34 points. FMA-UE was used in two different sets for each of the outcomes.

Two research physiotherapists, one in each study, performed the assessments according to the manuals for each assessment tool.

### Data Analysis

Descriptive statistics were used to present demographic data as well as stroke related variables, and are expressed in percentages, mean ± SD or median and interquartile range (IQR). The level of significance was set at *p* ≤ 0.05. The chi-square test and Mann-Whitney *U*-test were used to test for group differences in descriptive data. A logistic regression (37) was conducted to investigate whether FMA-UE was associated with impaired postural balance in stroke patients. Spearman's rank correlation coefficient (rho) was used to test correlations between the independent variables. The correlation values were interpreted as small (*r* < ±0.29), medium (*r* = ±0.30–0.49) or large (*r* = ±0.50–1.0) (38). Independent variables with a correlation coefficient <0.7 were entered into the regression analysis (39). Kendall's rank correlation test was used to test correlation between the dichotomised and independent variables, and a correlation coefficient >0.3 was required for inclusion in the regression analysis (39). To test the goodness of fit of the logistic regression models the Hosmer et al. (40) and the area under the curve (AUC) using Receiver Operating Characteristics Curves (ROC-curves) were performed. The results of the Hosmer et al. (40) were interpreted as poor fit (*p* < 0.05) or good fit (*p* >0.05), and the results of the AUC were classified as acceptable (AUC ≥ 0.7), splendid (AUC ≥0.8) or excellent (AUC ≥0.9).

Age, sex, stroke subtype according to the International Classification of Diseases (ICD) and time post-stroke were handled as possible confounders and were adjusted for in the regression models. In the classification of stroke subtype the subarachnoid hemorrhage (I60) and the intracerebral hemorrhage (I61) were merged to one group “hemorrhage.”

## Results

### Participants and Characteristics

Data from 121 patients were included in this study, 89 from GOTVED (24) and 32 from the study by Carvalho et al. (25). The mean age was 70 years (SD 12.3) and the majority were men (60%) (Table 1). Of the participants, 21% had impaired postural balance according to BBS and 27% according to TUG. Five patients were not able to perform the TUG and were dichotomised as having impaired postural balance. The patients with impaired postural balance were significantly older than the patients with not impaired postural balance in both BBS and TUG (*p* = 0.013/0.001), but there were no significant differences regarding sex, time post-stroke or stroke subtype (Tables 2, 3).

**Table 1:** Demographic and clinical characteristics of the study population.

**Characteristics**	**Participants**
Total, *n*	121
**Age, (y)**	
Mean (SD)	70.4 (12.3)
Median (IQR)	70 (62–80.5)
Sex, males (%)	72 (59.5)
**Stroke subtype**, ***n (%)***	
Ischemic/hemorrhage	108 (89.2)/13 (10.7)
**Time post-stroke for assessment (months)**	
Mean(SD)	25.2 (27.8)
Median (IQR)	12 (12–14.5)
FMA-UE, Median (IQR)	65 (58.5–66)
FMA-LE, Median (IQR)	34 (30–34)
BBS, Median (IQR)	52 (46–55)
TUG sec, Median (IQR)	11 (9–14.6)
**Assistive device**	
Cane/walker/wheelchair	12/5/2

*SD, standard deviation; IQR, interquartile range; FMA-UE, Fugl-Meyer Assessment scale Upper extremity; FMA-LE, Fugl-Meyer Assessments scale Lower extremity; BBS, Berg Postural balance Scale; TUG, Time Up and Go*.

**Table 2:** Comparison of characteristics between impaired postural balance (IPB) and not impaired postural balance (NIPB) groups according to BBS.

**Factors**	**IPB**	**NIPB**	***p*-value**
Total, *n*	25	96	
**Age (y)**			
Mean (SD)	76 (12.3)	69 (11.8)	
Median (IQR)	80 (63–87)	69 (61.7–79)	0.013
Sex, Male, *n* (%)	13 (52)	59 (62)	0.49
**Stroke subtype**, ***n*** **(%)**			
Ischemic/hemorrhage	21(84)/4(16)	87(91)/9(9)	0.46
**Time post-stroke for assessment (months)**			
Mean (SD)	27.7 (34.1)	24.5 (25.9)	0.635
Median (IQR)	12 (12–23)	12 (12–12.25)	
FMA-UE, Median (IQR)	58 (32–64)	65 (62–66)	<0.001
FMA-LE, Median (IQR)	31 (30–34)	34 (31–34)	0.001
TUG sec, Median (IQR)	21 (17–28)	10.3 (9–12.4)	<0.001

*SD, standard deviation; IQR, interquartile range; FMA-UE, Fugl-Meyer Assessment Upper extremity; FMA-LE, Fugl-Meyer Assessment Lower extremity; TUG, Time Up and Go*.

**Table 3:** Comparison of characteristics between impaired postural balance (IPB) and not impaired postural balance (NIPB) group according to TUG.

**Factors**	**IPB**	**NIPB**	***p*-value**
Total, *n*	33	88	
**Age (y)**			
Mean (SD)	76.7 (11.7)	67.9 (11.7)	
Median (IQR)	79 (66.5–86)	68 (61-78)	0.001
Sex, Male *n* (%)	15(45.5)	57 (64.8)	0.063
**Stroke subtype**, ***n*** **(%)**			
Ischemic infarct/Intracerebral hemorrhage	28/5	80/8	0.511
**Time post-stroke for assessment (months)**			
FMA-UE, Median (IQR)	60 (44.5–64)	65 (62–66)	<0.001
FMA-LE, Median (IQR)	31 (20.5–34)	34 (31.3–34)	<0.001
BBS, Median (IQR)	40 (31.5–46.5)	53 (51–56)	<0.001

*TUG, Time Up and Go; SD, standard deviation; IQR, interquartile range; FMA-U, Fugl-Meyer Assessment Upper extremity; FMA-LE, Fugl-Meyer Assessment Lower extremity; BBS, Berg Postural balance Scale*.

### Association Between Arm Function and Postural Balance

The correlation between motor function in the affected arm after stroke and postural balance was low when measured with BBS (rho 0.321, *p* < 0.001) and TUG (rho −0.315, *p* = 0.001), showing that better motor function in the arm measured with FMA-UE correlated with better postural balance (41). Having impaired motor function in the arm was significantly associated with impaired postural balance as assessed with BBS with OR = 0.879 (Table 4). Regression analysis with TUG showed the same result, OR = 0.868 for FMA-UE (Table 4). The regression model including motor function in the affected arm explained 49% (Nagelkerke R^2^ = 0.485) of the variance of postural balance with BBS as the dependent variable, and 54% (Nagelkerke R^2^ = 0.536) with TUG as the dependent variable (Table 4) showing that the TUG fits better to detect patients with impaired balance. Area under the ROC curve was 0.887 in the model with BBS and 0.888 with the model with TUG. This demonstrates that both models work well to detect patients with impaired balance.

**Table 4:** Adjusted estimates in logistic regression of factors associated with impaired postural balance assessed with BBS or TUG.

**Predictors of postural balance assessed with BBS**	**OR**	**95% CL**	***p*-value**
FMA-UE	0.879	0.826–0.934	<0.001
FMA-LE	0.707	0.601–0.832	<0.001
**Predictors of postural balance assessed with TUG**			
FMA-UE	0.868	0.813–0.927	<0.001
FMA-LE	0.633	0.513–0.782	<0.001

*Model with BBS: FMA-UE: Hosmer and Lemeshow test, p = 0.767. Area under the curve = 0.887. FMA-LE: Hosmer and Lemeshow test, p = 0.772. Area under the curve = 0.875. Model with TUG: FMA-UE: Hosmer and Lemeshow test, p = 0.722. Area under the curve = 0.888. FMA-LE: Hosmer and Lemeshow test, p = 0.204, Area under the curve = 0.889, n = 121. OR, odds ratio; CL, confidence interval; FMA-UE, Fugl-Meyer Assessment Upper extremity; FMA-LE, Fugl-Meyer Assessment Lower extremity n = 121*.

Of those with maximum outcome of FMA-LE, 37 (31%) had impaired motor function in the affected arm. Six of those 37 had impaired balance when measured with BBS and 8 when measured with TUG.

## Discussion

The present study shows that motor function in the affected arm significantly associates with impaired postural balance when assessed with BBS or TUG. To our knowledge, this is one of the first studies looking into this association. A study from 2001 concluded that muscle weakness is a factor that affects balance and can predict falls (42), but they don't specify if it means weakness in the upper or lower part of the body. As shown in two studies, an explanation could be that delays in sensory and motor conduction can cause changes to balance strategies and movement corrections (43, 44).

There are several different ideas surrounding the importance of the arm's motor skills and mobility when walking, losing postural balance etc. Some think that the arms may serve a protection function, reaching for external supports when you are about to fall (45). Others contend that arm movements serve as a counterweight to move the body's center of gravity away from the fall direction (46). Pijnappels et al. (22) concluded that the most important role that arm movement contributes toward postural balance recovery when falling is by changing the angle of the arm so that the person's center of gravity is displaced back toward equilibrium. During walking, arms normally swing in opposition to the legs, which helps to keep the COM within the BOS. When having a stroke with hemiparesis the UE the movements may be non- existing or minimal, and in general then locate the affected arm in front of their chest or beside the hip during walking. While the extent of the arm swing is limited or even non-existing, the patient is in danger of falling when walking (47). Boestrom et al. (48) noticed that movement of the upper body, in particular of the arms are not taken into account in several strategies of postural balance. Boestrom et al. (48) recently stated that when the arms were not actively held down, the upper body joints contribute substantially to balance regulation in the studied dynamic balance tasks, both with regard to torque amplitude and variation. In any case, current researchers agree that motor function and mobility in the arms play an important role for maintaining dynamic postural balance. Recent studies therefore suggest extending the concept of postural balance strategies, based on the assumption that the body is swaying around one joint, to include a multidimensional analysis including most major joints, such as, arm and trunk is warranted (49–51).

Our study shows that impaired motor function in the UE assessed with FMA-UE is associated with the presence of impaired postural balance in stroke patients. In the present study, 56% had maximum motor function in LE, and 30% had maximum motor function in UE assessed with FMA. Approximately one fifth of those with intact motor function in the lower extremity and impaired motor function in the affected arm had impaired postural balance. That makes this study particularly relevant to clinical practice. It can be of clinical importance to be aware of the fact that not only lower extremity impairment, but also arm function can have an impact on postural balance; and despite some patients having maximum motor function according to the FMA-LE their postural balance may still be affected by impaired arm function.

The difference in the prevalence of impaired postural balance in this study, depending on which outcome was used, may have resulted from the cut off we chose to use for each of the outcomes. The cut of that we adopted for the BBS also agreed quite well with a study from 2009 who found that to be community ambulating after a stroke you had to have a BBS >46.5 (32). The cut off for the TUG that we adopted agreed well with an earlier study, which concluded that with a TUG <14.8 elderly stroke patients were community ambulating (32). Both the studies regarding BBS and the two regarding TUG have the same conclusions regarding their cut off points, the higher risk for falls and being able to be community ambulating (29, 32, 33). Another possible reason for BBS correlating higher than TUG with postural balance could be that there are more dynamic items involved in BBS than in TUG. Nevertheless, the two outcomes did not really capture the same number of patients in our study. This also agrees with the Holmer and Lemeshow test, that supports the model demonstrating that TUG is slightly more worthwhile. However, at the same time the RUC curve supports both models being able to detect those patients with impaired postural balance.

This study presents some strong points. Firstly, we have quite a large group, 121 subjects, and the group corresponds to the total stroke group in Sweden according to Riksstroke data from 2018 regarding age, sex and stroke subtype (52). The Stroke Recovery and Rehabilitation Roundtable (SRRR) concluded that 6 months after stroke disease, one is considered to be in the chronic phase (23), which means that all the subjects in this study are in the same phase of recovery which can be seen as a strength in this study. All patients are community living at the time of assessment which must be seen as a strength. Another strength is that we have used assessment scales that have proved to be reliable and valid in elderly people and in patients with chronic stroke (27, 29, 53), and are commonly used in clinical and research settings as outcome measures in persons with stroke.

### Study Limitations

Some limitations of this study should be noted. One limitation could be the time of assessment. The majority of the participants, 74%, were assessed 1-year post stroke, while the average of the other 26% was 5 years post stroke. A limitation can also be that we have no knowledge of the participant's postural balance before stroke onset, which of course could have affected the results. Postural balance improvement post-stroke could be explained by the physical adaptations that might occur when individuals are back in the community performing ADL, but at the same time the risk of cormobidity burden and multimorbidity affects this group (54, 55), considering the average age in the study is 70 years. Often in chronic stroke, the impairments brought on by the initial injury can be compounded by secondary complications over time, compounders that together with the stroke impairments can affect postural balance negatively. Meaning that although some individuals maintain their fitness following a stroke, there are many who do not, which may have affected the postural balance in this study. Another limitation worth mentioning is that in this study we have not adjusted for comorbidity and stroke severity because we did not have access to that data. A final limitation in this study is that since the two studies were made in different settings and the first study 10 years before the second, no combined assessment training between the researchers were performed.

The findings in this study suggest that the rehabilitation of the upper extremity is important not just for improving the function of the arm but also for improving the balance after stroke. Earlier studies have shown that prediction of function in the upper extremity is possible very early after stroke (56). With the findings we gained through this study, the upper extremity function may also help us to predict reduced postural balance capacity and thereby help reduce the risk of falls post stroke. Further studies need to investigate the possibility of predicting postural balance by assessing arm function after stroke.

## Conclusions

In conclusion, impaired arm function is associated with problems with postural balance in a late stage after stroke. This can be of clinical importance and further research ought to be undertaken to investigate the association in the acute stage.

## Data Availability

All datasets generated for this study are included in the manuscript and/or the supplementary files.

## Ethics Statement

The Regional Ethics Examination Board in Gothenburg (042-11, 426-05) approved this study. Written informed consent was obtained from the participants or from their closest relative prior to participation in any of the studies.

## Author Contributions

All authors made substantial contributions to concept, design, acquisition, and interpretation of data. LR drafted the manuscript and AD and KS revised it critically for intellectual content. All authors read and approved the final manuscript.

### Conflict of Interest Statement

The authors declare that the research was conducted in the absence of any commercial or financial relationships that could be construed as a potential conflict of interest.

## Abbreviations

ADL: activity of daily living
BBS: Berg Balance Scale
CL: confidence interval
FMA: Fugl-Meyer Assessment
FMA-UE: Fugl-Meyer Assessment Upper Extremity
FMA-LE: Fugl–Meyer Assessment Lower Extremity
GOTVED: Gothenburg Very Early Supported Discharge
IQR: inter quartil range
LE: lower extremity
OR: odds ratio
ROC: receiver operating characteristics
SRRR: Stroke Recovery and Rehabilitation Roundtable
TUG: Timed Up and Go
UE: upper extremity
WHO: World Health Organization.
